# CHA_2_DS_2_-VA Score Predicts Mortality but Not Embolic Events in Left-Sided Infective Endocarditis

**DOI:** 10.3390/microorganisms14102186

**Published:** 2026-09-30

**Authors:** Andrea Oña, Javier López, María de Miguel-Álava, Isidre Vilacosta, Carmen Olmos, Carmen Sáez, Adrián Lozano, Gonzalo Cabezón, Pablo Zulet, Adrián Jerónimo, Daniel Gómez, Paloma Pulido, Itziar Gómez-Salvador, Daniel Pinilla-García, J. Alberto San Román

**Affiliations:** 1 Instituto de Ciencias del Corazón (ICICOR), Instituto de Investigación Biosanitaria de Valladolid (IBioVALL), Hospital Clínico Universitario, 47003 Valladolid, Spain; 2 Centro de Investigación Biomédica en Red de Enfermedades Cardiovasculares (CIBERCV), Instituto de Salud Carlos III, 28029 Madrid, Spain; 3 Instituto Cardiovascular, Hospital Clínico San Carlos, Instituto de Investigación Sanitaria del Hospital Clínico San Carlos (IdISSC), 28040 Madrid, Spain; 4 Departament of Medicine, Universidad Complutense de Madrid, 28040 Madrid, Spain; 5 Departament of Medicine, Universidad Europea de Madrid, 28670 Madrid, Spain; 6 Instituto de Investigación Sanitaria del Hospital de la Princesa (IIS-P), Hospital la Princesa, 28006 Madrid, Spain

**Keywords:** infective endocarditis, in-hospital mortality, embolic events, CHA_2_DS_2_-VA score, atrial fibrillation

## Abstract

The CHA_2_DS_2_-VASc score is widely used to predict thromboembolic events in non-valvular atrial fibrillation (AF) and has also been linked to adverse outcomes in diverse clinical contexts. The latest published European Guidelines on AF recommend the simplified CHA_2_DS_2_-VA score. Embolic events (EEs) develop in up to 50% of patients with left-sided infective endocarditis (LSIE). Although several variables have been associated with in-hospital mortality and EEs, no standardized risk stratification tool is currently applied in daily practice. Therefore, we sought to assess the prognostic value of the CHA_2_DS_2_-VA score at admission for predicting both in-hospital mortality and EEs in LSIE. This study included a total of 1420 episodes of definite LSIE diagnosed across three tertiary centers. The CHA_2_DS_2_-VA score was calculated on admission for all patients. Only cases of clinical EEs occurring in the context of LSIE diagnosis and confirmed by imaging techniques were considered. In-hospital mortality was 28.8% with a stepwise increase according to the CHA_2_DS_2_-VA score (*p* < 0.001), regardless of AF history. Multivariable analysis identified this score as an independent predictor of mortality (OR 1.195: 95% CI 1.106–1.291). During hospitalization, 27.5% of patients experienced EEs, but no significant association was observed with the CHA_2_DS_2_-VA score (*p* = 0.753). In conclusion, the CHA_2_DS_2_-VA score emerges as a simple and widely available tool to identify LSIE patients at increased risk of in-hospital mortality, although it does not predict EEs.

## 1. Introduction

The CHA_2_DS_2_-VASc score is a simple bedside tool routinely used in the prediction of thromboembolic events in patients with non-valvular atrial fibrillation (AF). This score has been recognized as a predictor of adverse outcomes in various clinical contexts, such as heart failure [1], transcatheter aortic valve implantation [2] and acute coronary syndromes [3,4]. The recently published European AF Guidelines now recommend the use of CHA_2_DS_2_-VA [5], in which female gender has been excluded.

Left-sided infective endocarditis (LSIE) is a severe disease associated with high morbidity and mortality, which has not declined in recent years despite advances in its diagnosis and treatment [6]. The in-hospital mortality rate of LSIE ranges from 15% to 30% [6] and is mostly attributed to three causes: heart failure, uncontrolled infection and embolic events (EEs). EEs mainly occur due to the migration of vegetation material through the systemic circulatory system. The incidence of EEs varies depending on whether asymptomatic EEs are considered. When these are included, up to 50% patients experience EEs during the course of LSIE [7] whereas clinically evident EEs occur in approximately 10–30% of patients with LSIE [8]. The most commonly affected organs are the brain and the spleen, although any location within the systemic circulation can be affected. The highest risk of EEs is within the first days following the initiation of antibiotic treatment [9]. Many variables have been associated [10], but vegetation size and prior embolism are the only parameters mentioned in the ESC and AHA guidelines as predictors of EEs [6,11]. To the best of our knowledge, very few studies have focused on the role of the CHA_2_DS_2_-VASc score and none of CHA_2_DS_2_-VA as predictors of prognosis in LSIE.

Our hypothesis was that in patients with LSIE, the CHA_2_DS_2_-VA score at admission might be associated with in-hospital mortality and EEs.

## 2. Materials and Methods

### 2.1. Population

Every patient with a definite LSIE according to the diagnostic criteria of the guidelines applicable at each time during the study period, admitted to three tertiary centers from 1994 to 2023 was included in an ongoing multipurpose database. Right-sided episodes were excluded because of their different epidemiology and characteristics. The CHA_2_DS_2_-VA score was calculated at admission in all patients, regardless of AF history.

### 2.2. Endpoints

The two primary endpoints were: (1) in-hospital mortality and (2) symptomatic EEs developed during hospitalization and before valve surgery if it was performed.

### 2.3. CHA_2_DS_2_-VA Score

The baseline CHA_2_DS_2_-VA score at the time of LSIE diagnosis was calculated according to the current guidelines of AF management. Based on this score, patients were given 1 point for previous events of congestive heart failure, arterial hypertension, age between 65 and 74 years, diabetes mellitus and vascular disease, and 2 points for age ≥75 years and previous stroke or transient ischemic attack.

Heart failure was considered only when documented as a pre-existing condition prior to the index hospitalization for LSIE and was included in the CHA_2_DS_2_-VA score accordingly. Heart failure occurring at admission as a manifestation of the current IE episode was not considered for the heart failure component of the score.

### 2.4. Definition of Embolic Events

For the purposes of this study, we only considered symptomatic EEs that occurred after the diagnosis of LSIE and were confirmed by imaging techniques. This definition was chosen to focus on clinically apparent embolic complications, as systematic screening for asymptomatic embolic lesions is not routinely recommended in all patients with IE [6]. Cutaneous manifestations were not included.

Only patients with adequate follow-up for outcome assessment were included in the univariable analysis of EEs. Of the 1420 patients in the cohort, 1266 were eligible for the analysis of this endpoint. The remaining 154 patients were excluded because EEs could not be reliably ascertained owing to cardiac surgery within the first 48 h after admission (*n* = 126), death within the first 48 h (*n* = 15), or missing follow-up data (*n* = 13).

### 2.5. Definitions of Clinical Events and Variables

For the purposes of this study, the main clinical variables, microbiological and imaging findings, and clinical outcomes were defined as follows. Nosocomial infection was defined as an infection acquired during hospitalization or associated with healthcare exposure. Degenerative valve disease was defined as pre-existing structural valvular disease of degenerative etiology, whereas congenital valve disease was pre-existing valvular disease resulting from a congenital cardiac abnormality. Prosthetic valve was IE involving a mechanical or bioprosthetic heart valve. Native aortic valve involvement was defined as infection in the native aortic valve. Mechanical mitral valve prosthesis was the affection of the mechanical prosthetic valve in the mitral position. Dental manipulation was defined as any invasive dental procedure performed within the 3 months preceding the diagnosis of IE. Indwelling intravascular catheter was defined as the presence of an intravascular catheter at the onset of symptoms or at the time of diagnosis of IE.

Acute onset of symptoms was defined as the presence of symptoms attributable to IE for less than 15 days before hospital admission. Referral from a non-tertiary center referred to patients transferred to our center after diagnosis of LSIE from a non-tertiary hospital or from a hospital without on-site cardiac surgery facilities.

Regarding microbiological findings, positive blood cultures at admission were defined as the isolation of a microorganism considered causative of IE from blood cultures obtained at or around hospital admission. Persistent positive blood cultures despite antibiotic therapy were those with continued growth of the causative microorganism in blood cultures obtained 48–72 h after initiation of appropriate antibiotic therapy. Negative culture IE was defined as the absence of identification of a causative microorganism in blood cultures throughout the diagnostic evaluation of IE, including the absence of microbiological identification from other diagnostic specimens, such as surgically obtained valvular or embolic tissue, when applicable. Polymicrobial infection was defined as the isolation of two or more microorganisms considered causative of the same episode of IE.

Echocardiographic findings were defined according to the criteria established in current international consensus documents and recommendations on cardiac imaging in IE. Pulmonary hypertension was documented by echocardiography or right-heart catheterization according to contemporary diagnostic criteria.

Regarding clinical manifestations at admission, embolism at admission was defined as clinical or imaging evidence of an embolic event attributable to IE occurring before hospital admission. Renal failure was identified as acute kidney injury present at admission according to established diagnostic criteria. Heart failure (HF) was diagnosed as a clinical syndrome consistent with HF present at admission, based on symptoms and signs of congestion and/or low cardiac output. Septic shock was defined as sepsis associated with persistent hypotension requiring vasopressor therapy and a serum lactate concentration >2 mmol/L despite adequate volume resuscitation.

Finally, regarding in-hospital outcomes, surgery was defined as cardiac surgery performed as treatment for IE during hospitalization. Persistent infection was defined as ongoing clinical, microbiological, or imaging evidence of active IE despite appropriate antimicrobial therapy.

### 2.6. Ethics

The study protocol was approved by local ethic committee of our hospitals (CINV 16-123). Informed consent to include the data in an ongoing multipurpose database was obtained during the index hospitalization. Due to the retrospective nature of the study, specific informed consent was not obtained.

### 2.7. Statistical Analysis

Continuous variables are reported as mean ± SD or median [interquartile range]. Assumption of normality was verified by the Kolmogorov–Smirnov test and q-q plots. Categorical variables are expressed with the absolute number and percentage. Comparisons between groups were made using the χ^2^ test or Fisher’s exact test for categorical variables when expected frequencies were less than 5. To measure and test the significance of the trend in the proportions of mortality and EEs as the CHA_2_DS_2_-VA score increases χ2 test for linear trend was used. For continuous variables, Student’s *t* test or U Mann–Whitney test was used.

A multivariable analysis by a logistic regression model with the maximum likelihood of using a backward stepwise method was assessed to analyze the prognostic factors of in-hospital mortality in LSIE. Sex, anemia, chronic bronchopathy, nosocomial IE, *Staphylococcus aureus*, periannular complication, prosthetic valve IE, mitral valve involvement, heart failure at admission, acute renal failure at admission, septic shock at admission, CHA_2_DS_2_-VA score, persistent infection, surgery and appropriate empiric antimicrobial therapy were included in the model.

For the adjusted model, the ratio variable/event was controlled to avoid overfitting. Odds ratios (OR) adjusted for each of the variables included, along with their 95% confidence intervals were calculated. Non-collinearity was checked among the variables. Calibration was evaluated with the Hosmer–Lemeshow test. The area under the receiver operating characteristic curve (ROC curve) was used to measure the discriminatory capacity.

Statistical analysis was performed using R software, version 4.3.3 (R Core Team, R Foundation for Statistical Computing, Vienna, Austria). All tests were two-sided. Differences were statistically significant when the *p*-value was <0.05.

## 3. Results

### 3.1. Baselines Characteristics

A total of 1420 episodes of definite LSIE were included in the study. The baseline characteristics of our population are shown in Table 1.

Male sex prevailed (65%), and the mean age at diagnosis was 65.7 ± 14.7 years. Most patients presented with community-acquired LSIE and 80.8% had positive blood cultures at admission. *Staphylococcus aureus* was the most prevalent microorganism, followed by *viridans* group *streptococci* and *Enterococcus* spp. Mortality rates differed significantly according to the causative microorganism, with the highest mortality observed in *S. aureus* infection (50.2%), whereas no significant differences were found in the incidence of EEs among the different microbiological groups (Table 2). The distribution of microorganisms, together with the corresponding mortality and embolic event rates, are summarized in Table 1 and Table 2.

When comparing baseline characteristics between survivors and patients who died during hospitalization, deceased patients were older and had a higher prevalence of nosocomial infection and comorbidities (diabetes mellitus, chronic bronchopathy and chronic real failure). They also presented a higher burden of echocardiographic complications related to IE, as well as a greater incidence of heart failure and septic shock at admission (Table 1). Overall, these findings indicate a more severe clinical profile among patients who died during hospitalization.

Heart failure signs and symptoms were documented in 38.7% of the patients at admission and 67% had significant valvular regurgitation. Cardiac surgery was required in 61% of patients.

**Figure 1:**
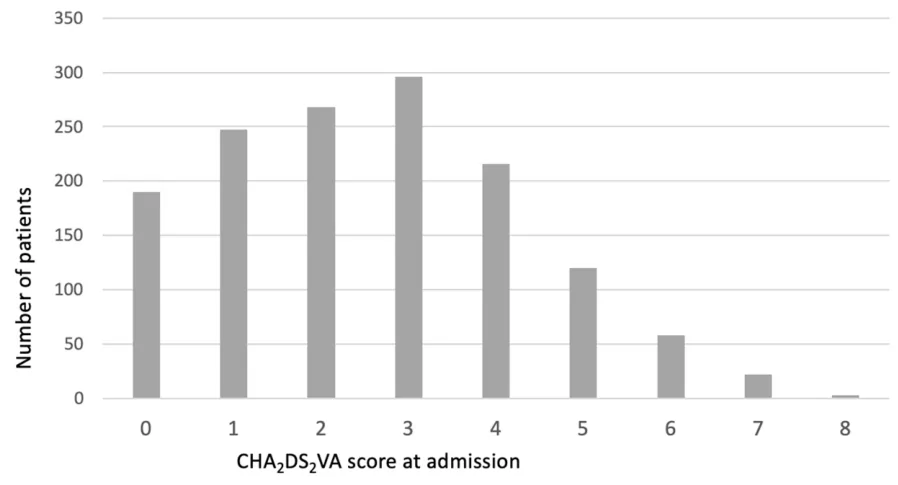
Distribution of patients according to CHA_2_DS_2_-VA score at admission.

The distribution of patients according to the CHA_2_DS_2_-VA score at admission is shown in Figure 1. The most prevalent score was 3 (n = 296) and the least prevalent were those higher than 4. Half of the patients had a score ≤2.

### 3.2. In-Hospital Mortality

**Figure 2:**
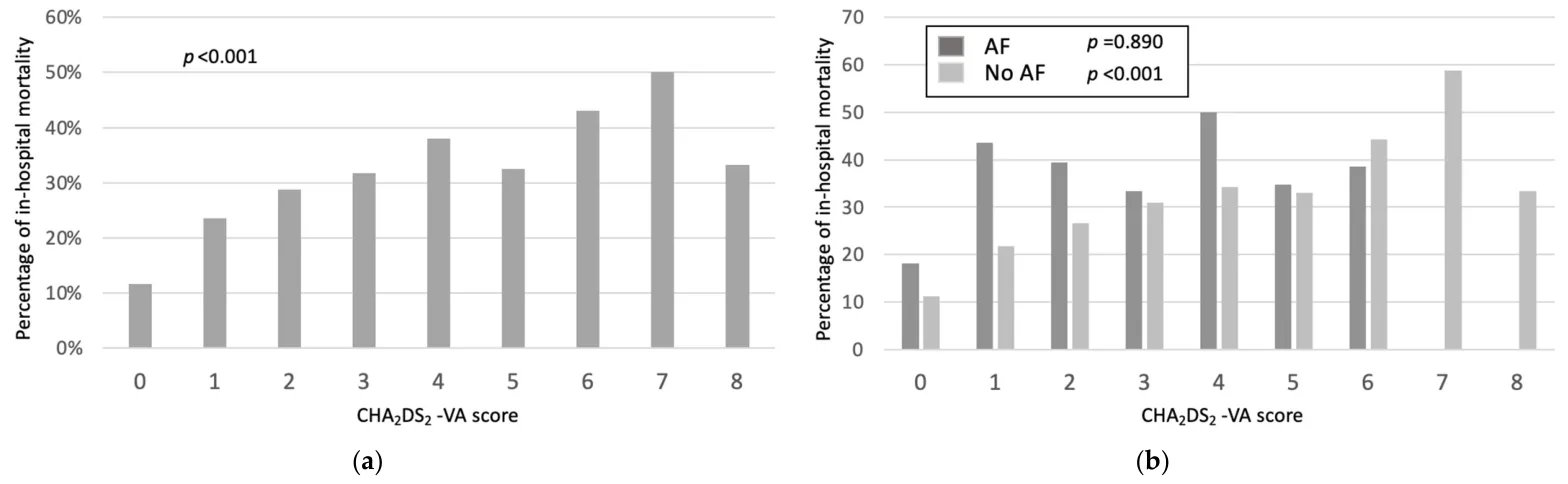
(**a**) Distribution of in-hospital mortality according to CHA_2_ DS_2_-VA score. (**b**) Distribution of in-hospital mortality according to CHA_2_DS_2_-VA score, stratified by the presence or absence of known atrial fibrillation (AF), with *p*-values for the association between CHA_2_DS_2_-VA score and in-hospital mortality in each subgroup.

The in-hospital mortality was 28.8% (n = 409). The main causes of death were: multiorgan failure (46%), septic shock (36.9%) and heart failure (31.5%). In-hospital mortality according to the CHA_2_DS_2_-VA score is represented in Figure 2. There were statistically significant differences in mortality according to the CHA_2_DS_2_-VA score (*p* <0.001). Accordingly, we performed an additional analysis evaluating in-hospital mortality across the individual CHA_2_DS_2_-VA scores. There was a progressive increase in the proportion of patients who died across increasing score values, from 11.6% among patients with a score of 0 to 50.0% among those with a score of 7. Although the number of patients with the highest scores was relatively small, mortality increased across the individual CHA_2_DS_2_-VA score values.

Because of the relatively small number of patients at the highest individual scores, we also evaluated clinically meaningful score categories. Using the predefined categories of score 0–1, 2–3, 4–5, and 6–8, in-hospital mortality increased progressively from 18.3% in patients with scores of 0–1 to 44.6% in those with scores of 6–8 (*p* < 0.001).

We also assessed in-hospital mortality according to CHA_2_DS_2_-VA score in patients with and without a history of AF. Among patients without known AF, mortality increased progressively across CHA_2_DS_2_-VA score categories, with a significantly higher mortality among patients with higher scores (*p* < 0.001). Among patients with known AF, mortality rates were higher in patients with higher CHA_2_DS_2_-VA scores overall, although the association did not reach statistical significance (*p* = 0.890). These findings are illustrated in Figure 2.

### 3.3. Embolic Events

Among the 1420 patients included in the overall cohort, 1266 had available information regarding EEs and were included in the EE analysis. In our cohort, the incidence of EEs during hospitalization was 27.5% (n = 348). The most affected organs were the central nervous system (48.2%), spleen (35.3%), kidneys (12.1%) and liver (4.2%).

**Figure 3:**
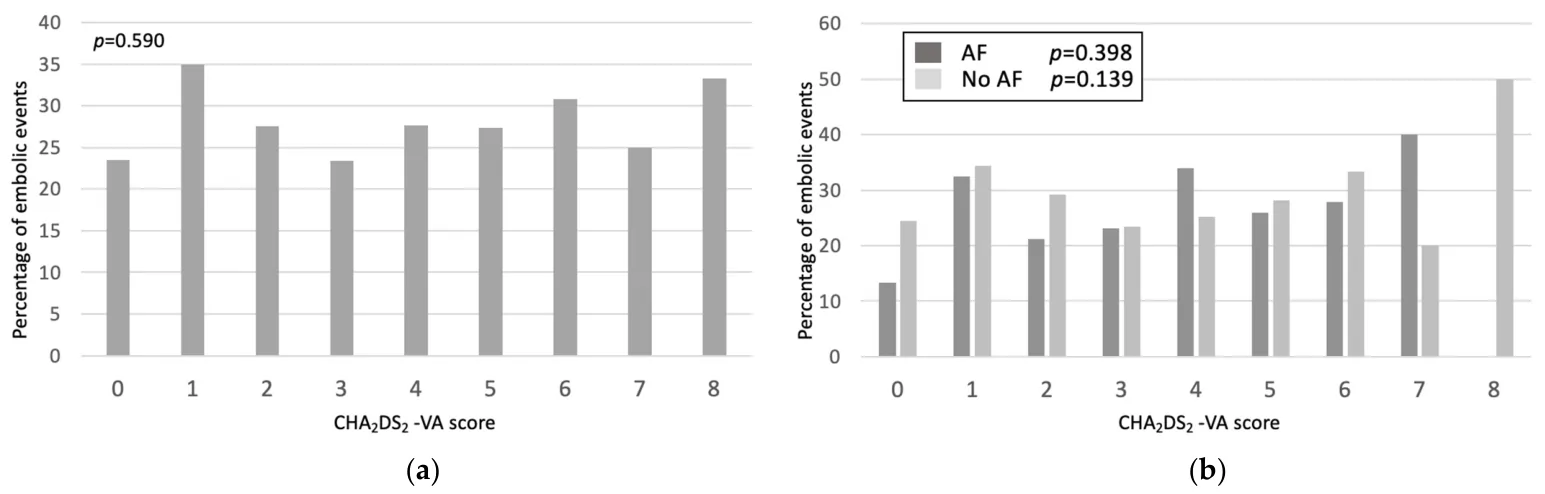
(**a**) Percentage of embolic events during hospitalization according to CHA_2_DS_2_-VA score at admission; (**b**) Percentage of embolic events during hospitalization according to CHA_2_DS_2_-VA score in patients with and without atrial fibrillation (AF).

There were no differences in the incidence of EEs based on the CHA_2_DS_2_-VA score (*p* = 0.590), neither in the total population nor in patients with or without AF (Figure 3). Similarly, no significant differences were observed when the embolism present at the time of diagnosis were included in the analysis (*p* = 0.151).

### 3.4. Predictors of Mortality

The univariable analysis of in-hospital mortality in the 1420 patients of our series is shown in Table 1.

**Figure 4:**
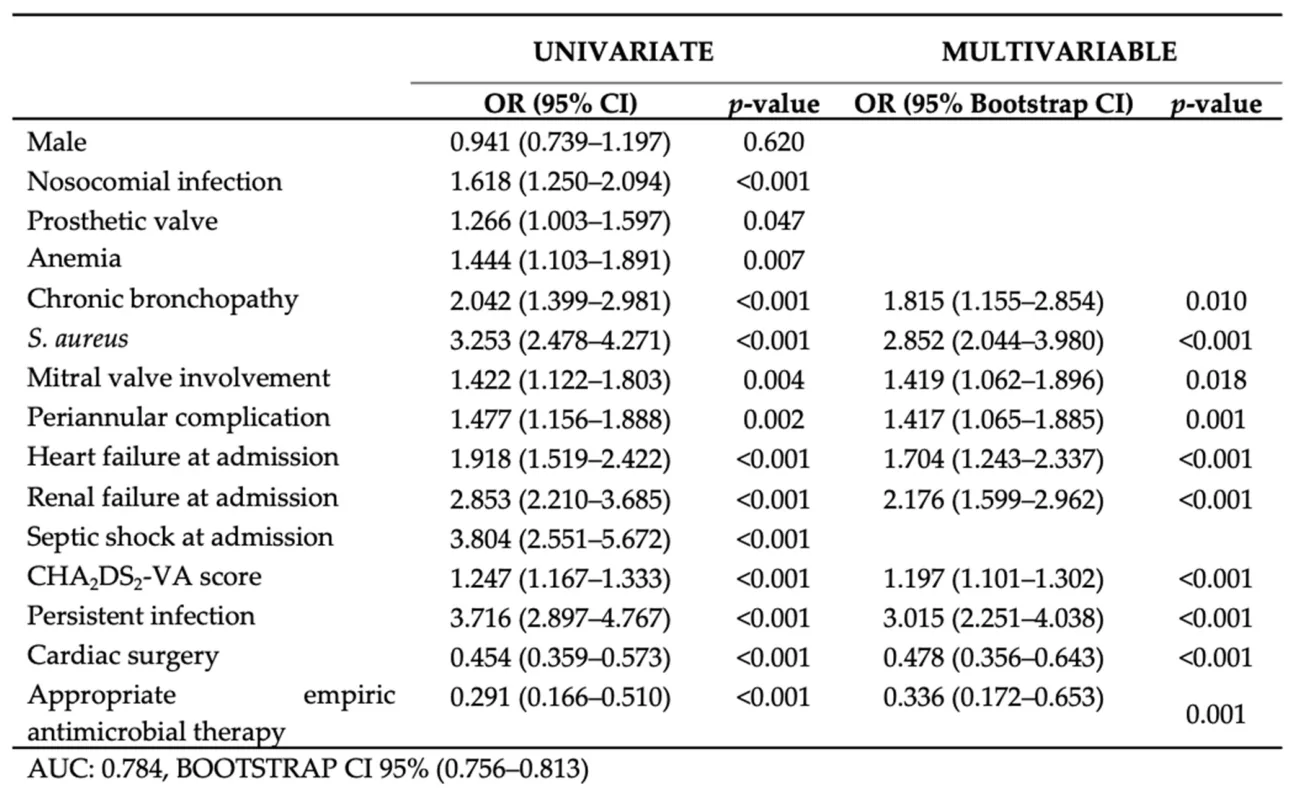
Univariable and multivariable analyses of predictors of in-hospital mortality. Odds ratios (ORs) and 95% confidence intervals (CIs) are presented for the univariable and multivariable logistic regression analyses.

To determine potential variables independently associated with LSIE mortality, a multivariable analysis was also performed. We included in the model variables that were significant and clinically relevant in the univariable analysis. Prognostic factors of mortality are shown in Figure 4. Of note, the CHA_2_DS_2_-VA score was independently associated with mortality [OR 1.197; 95% CI (1.101–1.302)].

### 3.5. Predictors of Embolic Events

To further investigate factors associated with the occurrence of EEs during hospitalization, a univariable analysis was performed. The associations between baseline clinical, microbiological, and echocardiographic characteristics and the occurrence of EEs are presented in Table 2**.** EEs were more frequently observed in patients with native mitral valve IE, acute symptom onset and the presence of vegetations on echocardiography.

**Figure 5:**
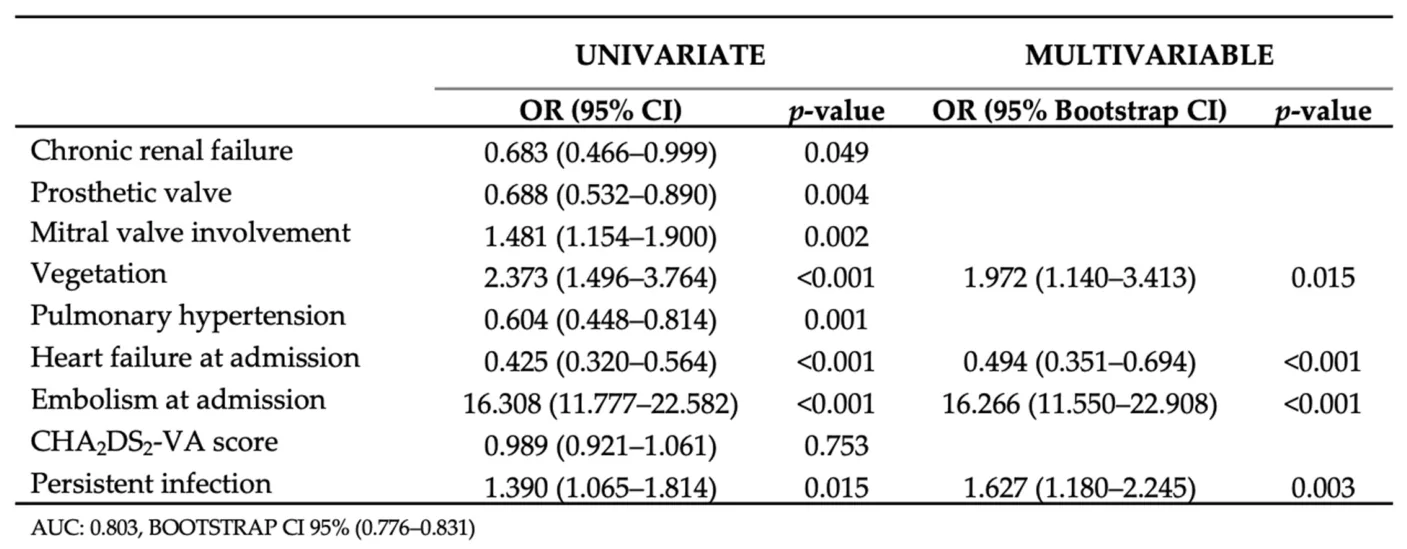
Univariable and multivariable analyses of predictors of embolic events. Odds ratios (ORs) and 95% confidence intervals (CIs) are presented for the univariable and multivariable logistic regression analyses. The CHA_2_DS_2_-VA score was included as a continuous variable.

A multivariable analysis was also conducted to identify variables independently associated with the occurrence of EEs during hospitalization. Variables showing statistical significance and clinical relevance in the univariable analysis were entered into the model. Independent predictors of EEs are presented in Figure 5. Additionally, the analysis was repeated using the CHA_2_DS_2_-VA score, with no changes observed in the results.

To assess the potential influence of embolisms present at admission on the model, a sensitivity analysis was performed after excluding these patients. In this analysis, vegetation, heart failure at admission and persistent infection remained independently associated with subsequent EEs, whereas CHA_2_DS_2_-VA score remained non-significantly associated with EEs (OR 1.061, 95% CI 0.980–1.149; *p* = 0.145).

## 4. Discussion

Our study shows that the CHA_2_DS_2_-VA score is independently associated with mortality but not with EEs in LSIE.

Despite medical advances in recent years, LSIE still bears a poor short-term outcome. Many studies have searched for high-risk prognostic variables with discordant results [12,13,14,15]. From a clinical point of view, it is difficult to calculate the risk for a particular patient, and it would be very useful to have easily available prognostic tools that could stratify patient risk.

The CHA_2_DS_2_-VASc score was developed to predict the cardioembolic risk in patients with non-valvular AF to initiate anticoagulant treatment. This score was also validated as a predictor of mortality in other cardiovascular scenarios, such as heart failure [1], transcatheter aortic valve implantation [2], in-stent restenosis [16] and acute coronary syndromes [3,4,17,18,19], irrespective of the cardiac rhythm. In the latest European AF guidelines [5] this score has been replaced by the CHA_2_DS_2_-VA score, which excludes the female gender variable. We have shown for the first time that the CHA_2_DS_2_-VA score is associated with higher mortality in patients with LSIE. One of the advantages of this score is its simplicity and the widespread familiarity with its use within the scientific community. Our results provide further evidence supporting the role of this score as a useful tool for risk stratification in patients with LSIE.

There have been two previous studies of the CHA_2_DS_2_-VASc score in IE. One group found that it is associated with in-hospital mortality [20]. Their population is quite different from ours, though, with a median age almost 10 years younger than in most of the previous studies, some patients had right sided IE and patients had lower risk (mortality rate 11.4%). Itelman et al. also concluded that the CHA_2_DS_2_-VASc score predicts in-hospital mortality [21]. They included right-sided IE too and their most frequent score was CHA_2_DS_2_-VASc 1 point, reflecting a less comorbid population than ours.

Regarding embolic risk, two scores have been developed. One included only *Staphylococcus aureus* etiology (OR 2.1) and vegetation size ≥ 13 mm (OR 2.1), with the presence of both factors increasing the 30-day embolic risk by almost 40% [22]. Hubert et al. also included these two parameters as well as some patients’ features such as age, diabetes, AF and previous embolism because those variables are considered prothrombotic conditions that might influence the formation and adhesion of thrombi to endothelial cells and increase embolic risk. However, they were not found to be significant in the univariate analysis [23].

In contrast to previous studies [24], we found no association between the causative microorganism, including *S. aureus*, and the occurrence of EEs. This may be explained by differences in the definition of EEs, as our analysis included only EEs occurring after hospital admission, whereas many previous studies also considered those events present at diagnosis. Following the initiation of antibiotic therapy, the risk of embolization declines rapidly and early surgery in selected high-risk patients may further reduce the incidence of subsequent EEs. Therefore, microbiological etiology may have a greater influence on EEs occurring before diagnosis than on those developing during hospitalization. In this regard, in a recent systematic review and metanalysis with more than 6000 patients, only vegetation size was associated with prognosis [25].

Our analysis was restricted to symptomatic EEs, as this endpoint has also been used in previous studies evaluating embolic risk in IE [23,26]. This approach may underestimate the overall burden of embolic lesions, as asymptomatic events can be detected by imaging; however, routine screening for silent embolism is not currently well established [6].

In our cohort, mitral valve involvement was associated with a higher risk of EEs, whereas prosthetic valve endocarditis was associated with a lower embolic risk. The first finding is consistent with previous studies and may be related to the greater mobility of mitral vegetations [24]. Conversely, the apparently protective effect of prosthetic valves should be interpreted with caution. A possible explanation is that prosthetic valve endocarditis more frequently presents with perivalvular complications, particularly prosthetic dehiscence [24], which often prompts early surgical intervention before EEs occur. Therefore, the lower incidence of EEs in this subgroup may reflect differences in clinical presentation and management rather than a lower embolic potential.

The CHA_2_DS_2_-VASc score is useful for assessing embolic risk even outside the context of AF. It has been described to predict the risk of stroke in patients who underwent transcatheter aortic valve implantation, both in the early post-procedural period and at the one-year follow-up. However, in the latter case, the association did not reach statistical significance [2]. According to our results, the CHA_2_DS_2_-VA score does not predict symptomatic EEs in LSIE. In line with these results, it was described that AF in LSIE increases mortality, as it leads to more heart failure decompensations. However, AF did not increase the risk of EEs or stroke in this setting [27]. These findings seem reasonable since the etiology of EEs in LSIE is not thrombotic. Therefore, our results do not support the use of this scale in this scenario.

## 5. Limitations

This study has several limitations that should be considered. Ours is an observational retrospective study, so we cannot rule out the presence of selection bias and unmeasured confounding factors. The restriction of the embolic endpoint to symptomatic EEs may have underestimated the overall burden of embolic lesions in LSIE. These findings should be validated in other populations. Our hospitals are tertiary care centers which receive severely ill patients from other hospitals. Thus, the CHA_2_DS_2_-VA score may not apply for other type of institutions.

## 6. Conclusions

In summary, in patients with LSIE, the CHA_2_DS_2_-VA score identifies patients with poor in-hospital outcome and might be used in the risk stratification process, but it is not useful to predict EEs.

## Figures and Tables

**Table 1:** Patients baseline characteristics of our study group. Values are presented as number (%) or mean ± standard deviation. Additionally, univariable analysis of predictors of mortality in LSIE is shown.

	Total (n = 1420)	Death (n = 409)	Alive (n = 1011)	*p*
Age	65.7 ± 14.7	70.1 ± 12.3	63.9 ± 14.9	<0.001
Male	934 (65.8%)	265 (64.8%)	669 (66.2%)	0.620
Nosocomial infection	344 (24.2%)	126 (30.8%)	218 (21.6%)	<0.001
Degenerative valve disease	310 (21.8%)	106 (25.9%)	204 (20.2%)	0.018
Congenital valve disease	74 (5.2%)	7 (1.7%)	67 (6.6%)	<0.001
Prosthetic valve	582 (41%)	184 (45%)	398 (39.4%)	0.053
Native aortic valve	485 (34.2%)	123 (30.1%)	362 (35.8%)	0.039
Mechanical mitral valve prosthesis	247 (17.4%)	85 (20.8%)	162 (16.0%)	0.032
Dental manipulation	93 (6.6%)	7 (1.7%)	86 (8.5%)	<0.001
Indwelling catheter	184 (13%)	75 (18.3%)	109 (10.8%)	<0.001
Diabetes mellitus	354 (24.9%)	129 (31.5%)	225 (22.3%)	<0.001
Anemia	307 (21.7%)	107 (26.3%)	200 (19.8%)	0.007
Chronic bronchopathy	122 (8.6%)	53 (13%)	69 (6.8%)	<0.001
Chronic renal failure	197 (13.9%)	89 (21.8%)	108 (10.7%)	<0.001
CHA_2_ DS_2_-VA score	3 [1–4]	3 [2–4]	2 [1–4]	<0.001
Acute onset of symptoms	730 (51.6%)	252 (62.1%)	478 (47.4%)	<0.001
Referral from a non-tertiary center	664 (46.8%)	200 (48.9%)	464 (45.9%)	0.304
Microbiology findings				
Positive blood cultures at admission	1071 (80.8%)	306 (80.5%)	765 (81%)	0.859
Persistent positive blood cultures despite antibiotic	228 (26%)	86 (35%)	142 (22.5%)	<0.001
Negative cultures	160 (11.3%)	47 (11.5%)	113 (11.2%)	0.870
*Streptococcus gallolyticus*	82 (5.8%)	13 (3.2%)	69 (6.8%)	0.008
Viridans group streptococci	240 (16.9%)	34 (8.3%)	206 (20.4%)	<0.001
*Enterococcus* spp.	201 (14.2%)	57 (13.9%)	144 (14.3%)	0.875
Other *Streptococcus* spp.	90 (6.3%)	18 (4.4%)	72 (7.1%)	0.056
*Staphylococcus aureus*	277 (19.5%)	139 (34%)	138 (13.7%)	<0.001
Other *Staphylococcus* spp.	241 (17%)	81 (19.8%)	160 (15.8%)	0.072
Gram-negative bacilli	61 (4.3%)	18 (4.4%)	43 (4.3%)	0.904
Fungi	35 (2.5%)	11 (2.7%)	24 (2.4%)	0.730
HACEK group	9 (0.6%)	1 (0.2%)	8 (0.8%)	0.460
Polymicrobial	68 (4.8%)	21 (5.1%)	47 (4.7%)	0.701
Other microorganisms	50 (3.5%)	6 (1.5%)	44 (4.4%)	0.007
Image findings				
Vegetation	1231 (86.7%)	371 (90.7%)	860 (85.1%)	0.005
Abscess	252 (17.7%)	86 (21%)	166 (16.4%)	0.040
Periannular complication	419 (29.5%)	145 (35.5%)	274 (27.1%)	0.002
Fistula	51 (3.6%)	27 (6.6%)	24 (2.4%)	<0.001
Pseudoaneurysm	227 (16.0%)	77(18.8%)	150 (14.8%)	0.063
Pulmonary hypertension	386 (27.3%)	143 (35.1%)	243 (24.2%)	<0.001
Symptoms at admission				
Embolism at admission	297 (21%)	89 (21.8%)	208 (20.7%)	0.643
Renal failure	340 (24%)	158 (38.6%)	182 (18.1%)	<0.001
Heart failure	548 (38.7%)	204 (49.9%)	344 (34.2%)	<0.001
Septic shock	109 (7.7%)	63 (15.4%)	46 (4.6%)	<0.001
Stroke	165 (11.7%)	54 (13.2%)	111 (11.0%)	0.247
Outcome				
Cardiac surgery	866 (61.0%)	194 (47.4%)	672 (66.5%)	<0.001
Persistent infection	413 (30.3%)	202 (50.9%)	211 (21.8%)	<0.001
Embolic events	348 (27.5%)	119 (32.2%)	229 (25.6%)	0.017

**Table 2:** Patients baseline characteristics of our study group. Values are presented as number (%) or mean ± standard deviation. Additionally, univariable analysis of predictors of embolic events in LSIE is shown.

	Total (n = 1266)	Embolic Events (n = 348)	No Embolic Events (n = 918)	*p*
Age	65.7 ± 14.7	66.1 ± 14.3	66.1 ± 14.4	0.955
Male	829 (65.5%)	235 (67.5%)	594 (64.7%)	0.346
Nosocomial infection	305 (24.1%)	74 (21.3%)	231 (25.2%)	0.155
Degenerative valve disease	282 (22.3%)	67 (19.3%)	215 (23.4%)	0.117
Congenital valve disease	65 (5.1%)	19 (5.5%)	46 (5.0%)	0.738
Prosthetic valve	515 (40.7%)	119 (34.3%)	396 (43.1%)	0.004
Native mitral valve	502 (39.7%)	162 (46.6%)	340(37.0%)	0.002
Mechanical mitral valve prosthesis	221 (17.5%)	48 (13.8%)	173 (18.8%)	0.034
Dental manipulation	84 (6.6%)	26 (7.5%)	58 (6.3%)	0.454
Indwelling catheter	156 (12.3%)	29 (8.4%)	127 (13.8%)	0.008
Diabetes mellitus	315 (24.9%)	90 (25.9%)	225 (24.5%)	0.601
Anemia	279 (22.1%)	71(20.5%)	208 (22.7%)	0.390
Chronic bronchopathy	103 (8.2%)	30 (8.7%)	73 (8.0%)	0.681
Chronic renal failure	178 (14.1%)	38 (11%)	140 (15.3%)	0.049
CHA_2_ DS_2_-VA score	3 [1–4]	3 [1–5]	2 [1–5]	0.590
Acute onset of symptoms	649 (51.6%)	196 (56.5%)	453 (49.6%)	0.029
Microbiology findings				
Positive blood cultures at admission	975 (81.5%)	280 (83.3%)	695 (80.8%)	0.313
Persistent positive blood cultures despite antibiotic	208 (26.1%)	58 (26.7%)	150 (25.6%)	0.804
Negative cultures	144 (11.4%)	38 (10.9%)	106 (11.6%)	0.749
*S. gallolyticus*	82 (5.8%)	24 (6.9%)	50 (5.5%)	0.328
Viridans group streptococci	240 (16.9%)	66 (19.0%)	156 (17.0%)	0.415
*Enterococcus* spp.	201 (14.2%)	53 (15.2%)	125 (13.6%)	0.465
Other *Streptococcus* spp.	90 (6.3%)	19 (5.5%)	65 (7.1%)	0.299
*Staphylococcus aureus*	277 (19.5%)	74 (21.3%)	178 (19.4%)	0.461
Other *Staphylococcus* spp.	241 (17%)	53 (15.2%)	155 (16.9%)	0.473
Gram-negative bacilli	61 (4.3%)	15 (4.3%)	42 (4.6%)	0.836
Fungi	35 (2.5%)	9 (2.6%)	17 (1.9%)	0.412
HACEK group	9 (0.6%)	4 (1.1%)	5 (0.5%)	0.270
Polymicrobial	68 (4.8%)	17 (4.9%)	44 (4.8%)	0.949
Other microorganisms	50 (3.5%)	9 (2.6%)	34 (3.7%)	0.326
Image findings				
Vegetation	1111 (87.8%)	325 (93.4%)	786 (85.6%)	<0.001
Abscess	223 (17.6%)	61 (17.5%)	162 (17.6%)	0.961
Periannular complication	369 (29.1%)	111 (31.9%)	258 (28.1%)	0.185
Fistula	42 (3.3%)	12 (3.4%)	30 (3.3%)	0.873
Pseudoaneurysm	202 (16.0%)	60 (17.2%)	142 (15.5%)	0.442
Pulmonary hypertension	340 (27.0%)	70 (20.2%)	270 (29.6%)	0.001
Symptoms at admission				
Embolism at admission	265 (20.9%)	197 (56.6%)	68 (7.4%)	<0.001
Renal failure	302 (23.9%)	83 (23.9%)	219 (23.9%)	0.998
Heart failure	454 (35.9%)	79 (22.7%)	375 (40.8%)	<0.001
Septic shock	84 (6.6%)	27 (7.8%)	57 (6.2%)	0.323
Stroke	148 (11.7%)	94 (27.0%)	54 (5.9%)	**<0.001**
Outcome				
Cardiac surgery	740 (58.5%)	205 (58.9%)	535 (58.3%)	0.855
Persistent infection	379 (30.9%)	121 (36.1%)	255 (28.9%)	**0.015**
Mortality	370 (29.2%)	119 (34.2%)	251 (27.3%)	**0.017**

## Data Availability

The data presented in this study are available on request from the corresponding author. The data are not publicly available due to privacy and ethical restrictions.

## Abbreviations

The following abbreviations are used in this manuscript:

AF	Atrial fibrillation
EEs	Embolic events
LSIE	Left-sided infective endocarditis
